# Racemization of the Succinimide Intermediate Formed in Proteins and Peptides: A Computational Study of the Mechanism Catalyzed by Dihydrogen Phosphate Ion

**DOI:** 10.3390/ijms17101698

**Published:** 2016-10-10

**Authors:** Ohgi Takahashi, Ryota Kirikoshi, Noriyoshi Manabe

**Affiliations:** Faculty of Pharmaceutical Sciences, Tohoku Medical and Pharmaceutical University, 4-4-1 Komatsushima, Aoba-ku, Sendai 981-8558, Japan; kirikoshi@tohoku-mpu.ac.jp (R.K.); manabe@tohoku-mpu.ac.jp (N.M.)

**Keywords:** succinimide, racemization, aspartic acid residue, nonenzymatic reaction, buffer catalysis, dihydrogen phosphate ion, enolization, proton transfer, computational chemistry, density functional theory

## Abstract

In proteins and peptides, d-aspartic acid (d-Asp) and d-β-Asp residues can be spontaneously formed via racemization of the succinimide intermediate formed from l-Asp and l-asparagine (l-Asn) residues. These biologically uncommon amino acid residues are known to have relevance to aging and pathologies. Although nonenzymatic, the succinimide racemization will not occur without a catalyst at room or biological temperature. In the present study, we computationally investigated the mechanism of succinimide racemization catalyzed by dihydrogen phosphate ion, H_2_PO_4_^−^, by B3LYP/6-31+G(d,p) density functional theory calculations, using a model compound in which an aminosuccinyl (Asu) residue is capped with acetyl (Ace) and NCH_3_ (Nme) groups on the N- and C-termini, respectively (Ace–Asu–Nme). It was shown that an H_2_PO_4_^−^ ion can catalyze the enolization of the H_α_–C_α_–C=O portion of the Asu residue by acting as a proton-transfer mediator. The resulting complex between the enol form and H_2_PO_4_^−^ corresponds to a very flat intermediate region on the potential energy surface lying between the initial reactant complex and its mirror-image geometry. The calculated activation barrier (18.8 kcal·mol^−1^ after corrections for the zero-point energy and the Gibbs energy of hydration) for the enolization was consistent with the experimental activation energies of Asp racemization.

## 1. Introduction

**Scheme 1:**
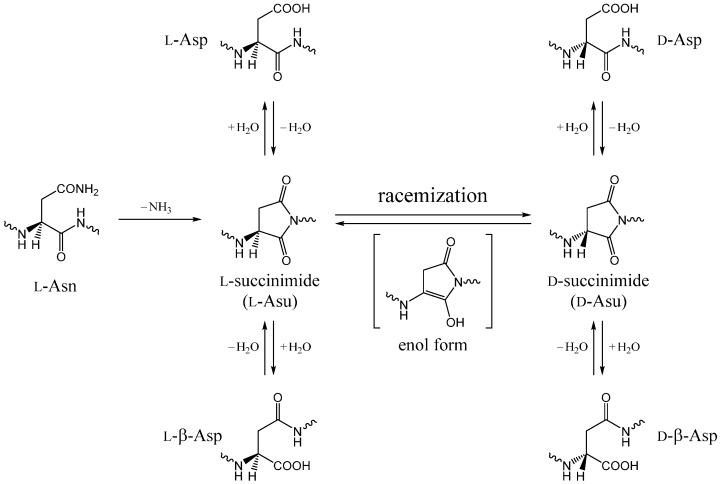
Succinimide-mediated, nonenzymatic reactions from l-Asp and l-Asn residues (Asu: aminosuccinyl residue). The succinimide formation from l-Asn is irreversible; the other reactions are reversible. At around pH 7, the succinimide racemization is expected to occur through an enol intermediate.

Except for glycine, the all α-amino acids used in protein biosynthesis are optically active and in the l-form. However, certain amino acid residues in proteins and peptides are prone to racemization, and these are mostly aspartic acid (Asp) residues. This is due to the formation of the five-membered ring succinimide intermediate (aminosuccinyl residue, Asu), which is the actual species undergoing direct racemization (Scheme 1) [1,2,3,4,5]. The five-membered ring is usually regarded to be formed by nucleophilic attack of the backbone nitrogen of the C-terminal neighboring residue on the Asp side-chain C_γ_ atom [6,7,8,9]. Hydrolysis of the succinimide intermediate leads either back to the original l-Asp residue or to the l-β-Asp residue, typically in a ratio of 1:3 [1,2,10,11,12,13,14,15]. Similarly, both d-Asp and d-β-Asp residues can be formed from the d-form of the succinimide intermediate. Succinimide intermediates can also be formed from asparagine (Asn) residues (this is irreversible because of the release of an ammonia molecule) [1,2,10,11,12,13,14]; therefore, in proteins and peptides, there can be d-Asp and d-β-Asp residues originating from l-Asn residues. The formation of the biologically uncommon l-β-Asp, d-Asp, and d-β-Asp residues have been paid considerable attention due to the relevance to aging and pathologies, especially those of age-related diseases such as cataract and Alzheimer’s disease [5,16,17,18,19,20,21,22,23,24,25,26,27,28,29,30,31,32,33,34,35,36,37,38,39,40,41,42].

Although the above succinimide-linked reactions in proteins and peptides are nonenzymatic, they will not occur at room or biological temperature without a catalyst because of high activation barriers [43,44,45,46,47,48,49]. Although buffer species are possible catalysts, we have little systematic insight into their possible catalytic roles. Tomizawa and co-workers [50] showed that, in hen egg white lysozyme, formation of d-Asp from l-Asp and/or l-Asn was accelerated by phosphate ions at pH 6 and 100 °C, suggesting that racemization of the succinimide intermediate is catalyzed by phosphate ions. At pH 6, phosphate ions exist mostly as dihydrogen phosphate, H_2_PO_4_^−^. At physiological pH of 7.4, this ion is also present in a significant amount.

**Figure 1:**
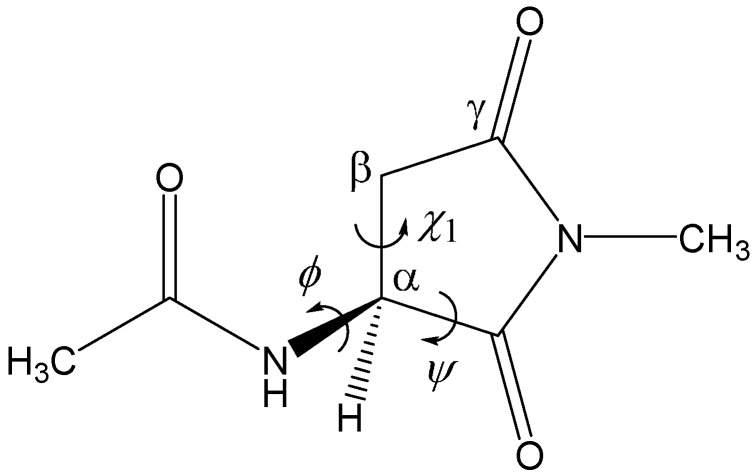
The model compound used in the present study (Ace–Asu–Nme, where Ace = acetyl, Asu = aminosuccinyl, and Nme = NCH_3_). The *φ* (C–N–C_α_–C) and *ψ* (N–C_α_–C–N) dihedral angles which characterize the main-chain conformation, and the *χ*_1_ dihedral angle (N–C_α_–C_β_–C_γ_) which characterizes the side-chain conformation are indicated. The Asu residue is in the l-configuration.

In the present paper, the possibility that the H_2_PO_4_^−^ ion catalyzes the racemization of Asu residues formed in proteins and peptides was computationally investigated by B3LYP/6-31+G(d,p) density functional theory (DFT) calculations. We used a model compound in which an Asu residue is capped with acetyl (Ace) and NCH_3_ (Nme) groups on the N- and C-termini, respectively (Ace–Asu–Nme, Figure 1). Only the extended backbone conformation was considered.

Stereoinversion at the Asu α-carbon atom (sp^3^-hybridized) requires that it becomes sp^2^-hybridized. Although a neutral enol (Scheme 1) and an enolate ion are possible candidates for an intermediate, the enolate ion will not be stable unless the pH is sufficiently basic. Here, it should be noted that the keto-enol tautomerism of aldehydes has been shown to be catalyzed by phosphate buffer (pH 7.4, 35 °C) [51]. Nitro-aci-nitro tautomerism is also catalyzed by phosphate buffer [52]. We show here that the H_2_PO_4_^−^ ion can catalyze the elolization of the H_α_–C_α_–C=O portion of Asu by acting as a proton-transfer mediator.

## 2. Results and Discussion

**Figure 2:**
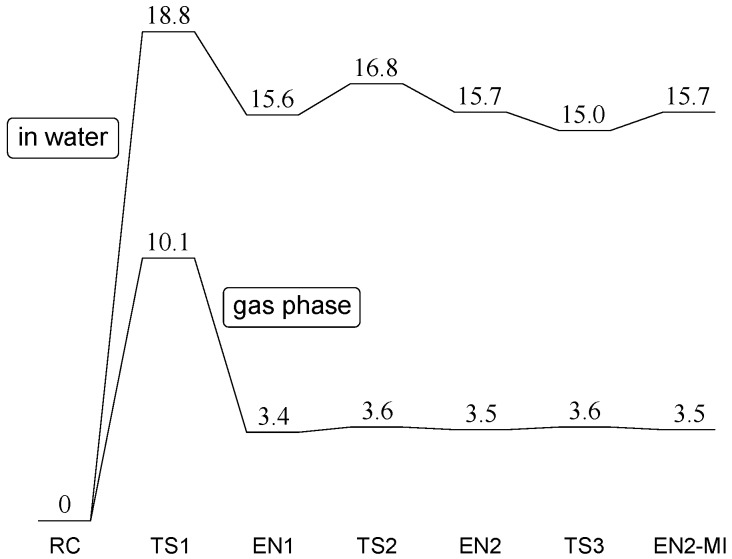
Energy profiles in the gas phase and in water. RC: reactant complex; TS: transition state; EN: enol; MI: mirror image. Relative energies with respect to the reactant complex (RC) are shown in kcal·mol^−1^ (1 cal = 4.184 J).

**Figure 3:**
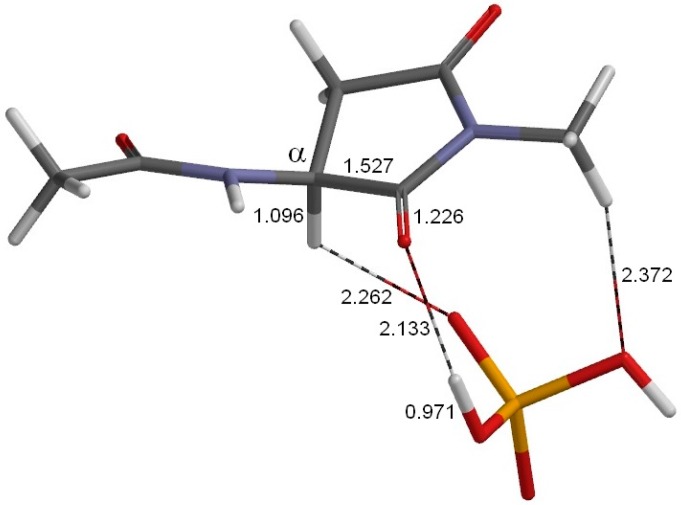
The geometry of the reactant complex RC (*φ* = 179°, *ψ* = −137°, *χ*_1_ = 131°). This complex is formed between the model compound (Figure 1) and an H_2_PO_4_^−^ ion. Selected interatomic distances are shown in Å. Grey: carbon; white: hydrogen; blue: nitrogen; red: oxygen; orange: phosphorus.

**Figure 4:**
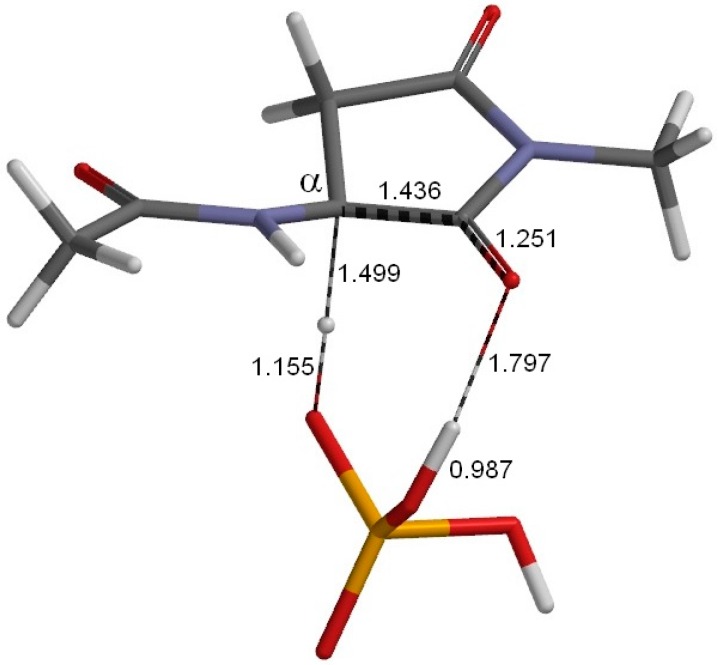
The geometry of the first transition state TS1 (*φ* = 178°, *ψ* = −150°, *χ*_1_ = 146°). This transition state is for enolization and connects RC (Figure 3) and EN1 (Figure 5). Selected interatomic distances are shown in Å. The imaginary frequency of this transition state is 856i cm^−1^. Grey: carbon; white: hydrogen; blue: nitrogen; red: oxygen; orange: phosphorus.

**Figure 5:**
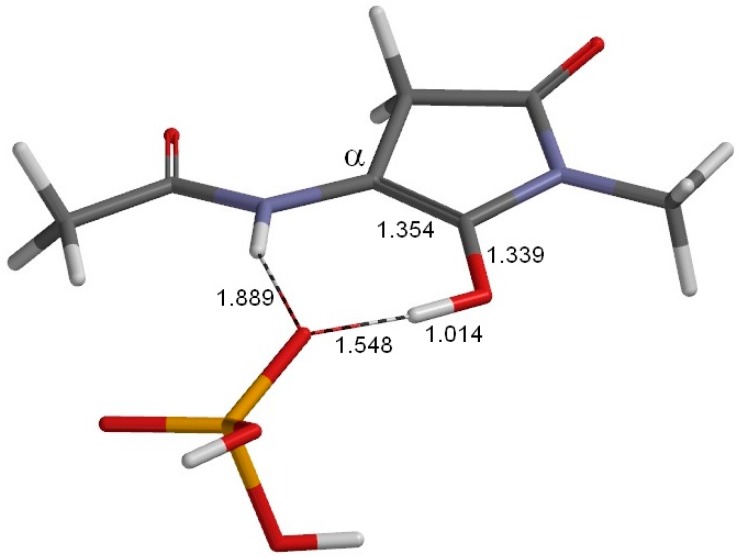
The geometry of the first enol minimum EN1 (*φ* = −165°, *ψ* = −177°, *χ*_1_ = 177°). This is a complex between the enol form of the model compound and an H_2_PO_4_^−^ ion, and lies between TS1 (Figure 4) and TS2 (Figure 6). Selected interatomic distances are shown in Å. Grey: carbon; white: hydrogen; blue: nitrogen; red: oxygen; orange: phosphorus.

**Figure 6:**
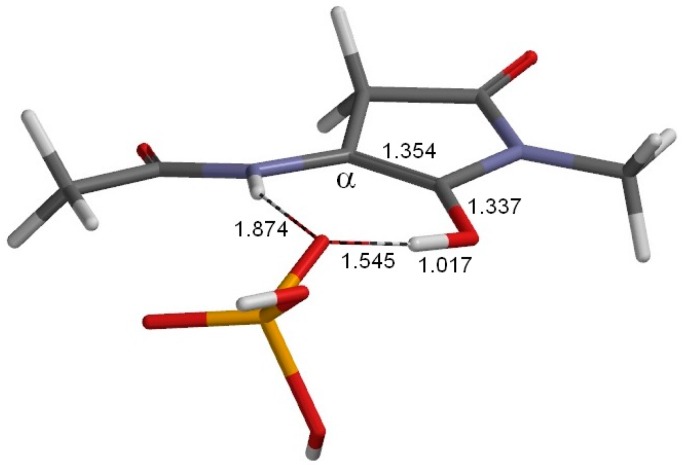
The geometry of the second transition state TS2 (*φ* = −160°, *ψ* = −176°, *χ*_1_ = 176°). This transition state connects EN1 (Figure 5) and EN2 (Figure 7), and is for an OH rotation in the H_2_PO_4_^−^ ion of the enol-H_2_PO_4_^−^ complex. Selected interatomic distances are shown in Å. The imaginary frequency of this transition state is 131i cm^−1^. Grey: carbon; white: hydrogen; blue: nitrogen; red: oxygen; orange: phosphorus.

**Figure 7:**
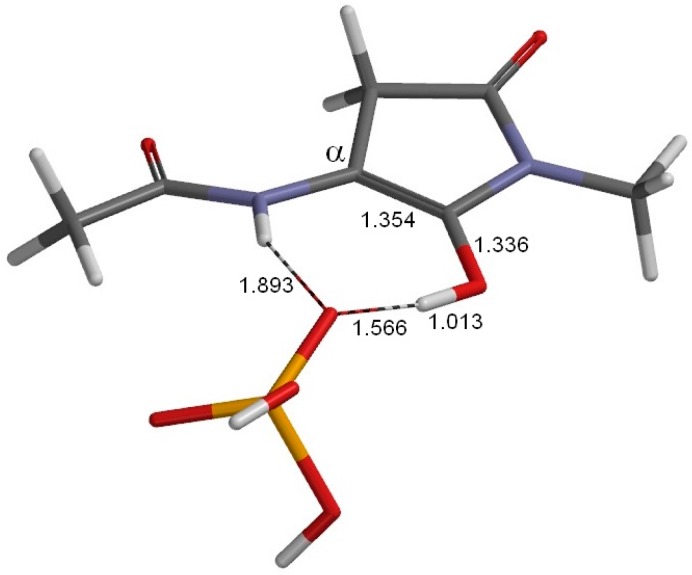
The geometry of the second enol minimum EN2 (*φ* = −159°, *ψ* = −177°, *χ*_1_ = 177°). This is related to EN1 (Figure 5) by an OH rotation in H_2_PO_4_^−^. Selected interatomic distances are shown in Å. Grey: carbon; white: hydrogen; blue: nitrogen; red: oxygen; orange: phosphorus.

**Figure 8:**
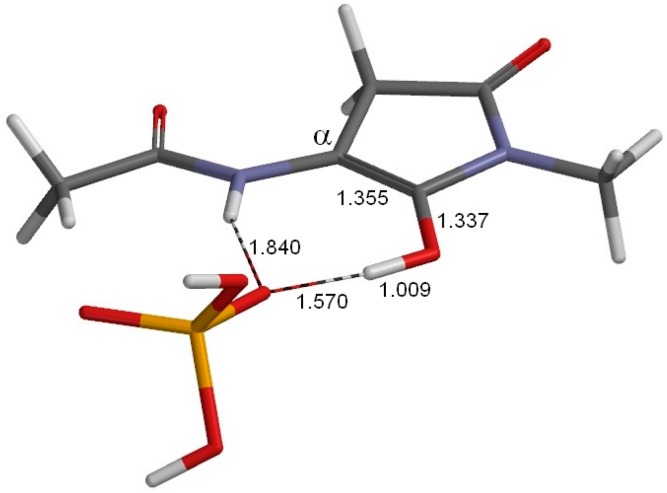
The geometry of the third transition state TS3 (*φ* = −169°, *ψ* = −177°, *χ*_1_ = 177°). This transition state connects EN2 (Figure 7) and its mirror-image geometry (EN2-MI). Selected interatomic distances are shown in Å. The imaginary frequency of this transition state is 18i cm^−1^. Grey: carbon; white: hydrogen; blue: nitrogen; red: oxygen; orange: phosphorus.

Figure 2 shows the energy profiles in the gas phase and in water, and Figure 3, Figure 4, Figure 5, Figure 6, Figure 7 and Figure 8 show the optimized geometries. Geometry optimizations were performed in the gas phase using the B3LYP functional and the 6-31+G(d,p) basis set. Gibbs energies of hydration were calculated for the gas-phase optimized geometries by the SM8 (solvation model 8) continuum model [53,54]. Relative energies were corrected for the zero-point energy (ZPE) calculated in the gas phase. All the minima connected by a given transition state (TS) were confirmed by intrinsic reaction coordinate (IRC) calculations followed by full geometry optimizations. The Cartesian coordinates, total energies, ZPEs, and SM8 Gibbs energies of hydration of the optimized geometries are provided in Tables S1–S7.

Figure 3 shows the geometry of the reactant complex (RC) formed between the model compound (in which the Asu residue is in the l-configuration) and an H_2_PO_4_^−^ ion. Although only the extended conformation was considered for the model compound, we actually found five energy minima for complexes with an H_2_PO_4_^−^ ion. The complex shown in Figure 3 (RC) is the most stable one when the hydration effect is included. In RC, a hydrogen bond (2.133 Å) is formed between the oxygen of the α-carbonyl group of Asu and one of the OH groups in H_2_PO_4_^−^. Moreover, two CH–O interactions are observed between the model compound and H_2_PO_4_^−^. One involves the Asu α-proton (2.262 Å), and the other (2.372 Å) involves the C-terminal methyl group. In the present model compound, the methyl proton relevant to the CH–O interaction mimics the α proton of the neighboring amino acid residue on the C-terminal side in actual proteins and peptides.

In Figure 2, the transition states are consecutively numbered as TS1, TS2, and TS3. RC is converted to the first enol minimum (EN1) via TS1, which is then converted to the second enol minimum (EN2) via TS2. EN2-MI stands for the mirror-image geometry of EN2, and TS3 is the transition state which connects EN2 and EN2-MI. The mirror-image geometry of RC, which contains a d-Asu residue, can then be reached via the mirror-image geometries of TS2, EN1, and TS1 consecutively.

Figure 4 shows the geometry of TS1, which is for the enolization of the H_α_–C_α_–C=O portion and connects RC and EN1. An extensive bond reorganization occurs in an eight-membered cyclic structure via TS1. In addition to the exchange of single and double bonds in the H_α_–C_α_–C=O portion, proton transfer from C_α_ to the carbonyl oxygen is required to reach the enol form. In the present mechanism, the H_2_PO_4_^−^ ion mediates this proton transfer. More specifically, the oxygen, which was weakly bound to H_α_, abstracts this proton, and the carbonyl oxygen abstracts a proton from H_2_PO_4_^−^. This double proton transfer is concerted but asynchronous, as may be seen from Figure 4; the former transfer precedes the latter.

Figure 5 shows the geometry of EN1. Because the C_α_ atom is now sp^2^-hybridized, the overall geometry of the enol isomer is close to planar (*ψ* = −177°). It is important to note that the chirality of the reactant is lost as soon as EN1 is reached. The H_2_PO_4_^−^ ion now forms two hydrogen bonds by one oxygen atom: one to the enol OH group (1.548 Å), and the other to the main-chain NH group (1.889 Å).

In water, the relative energies of TS1 and EN1 with respect to RC are 18.8 and 15.6 kcal·mol^−1^, respectively (1 cal = 4.184 J). These values are much higher than the corresponding values in the gas phase as shown in Figure 2. The large stabilization of RC in water may be due to a negatively-charged oxygen atom in H_2_PO_4_^−^ which is exposed to “bulk water”. The activation barrier of 18.8 kcal·mol^−1^ is reasonable for a reaction which proceeds under physiological conditions.

We also found a three-step route from EN1 to its mirror-image geometry (EN1-MI, geometry not shown). First, rotation of one of the OH groups in H_2_PO_4_^−^ proceeds, via TS2 (Figure 6), to give EN2 (Figure 7). EN2 is converted to its mirror-image geometry (EN2-MI, geometry not shown) via TS3 (Figure 8). EN2-MI can be converted to EN1-MI via the transition state corresponding to the mirror image of TS2 (TS2-MI, geometry not shown). The potential energy surface between EN1 and EN1-MI is extremely flat in the gas phase. Indeed, the imaginary frequency of TS3 is only 18i cm^−1^, and its energy became lower than that of EN2 and EN2-MI after correction for the Gibbs energy of hydration. We may say that there is a broad and flat enol intermediate region between EN1 and EN1-MI. It should also be noted that TS3 has *C*_1_ symmetry (not *C*_s_ symmetry). This means that there is another path connecting EN2 and EN2-MI, which passes through the mirror-image geometry of TS3 (TS3-MI, geometry not shown).

Finally, ketonization of EN1-MI via the mirror-image geometry of TS1 (TS1-MI, geometry not shown) results in stereoinversion of the original l-Asu residue. Note that, since our model compound has only one chiral carbon atom, the final product complex includes the enantiomer of the model compound.

As far as we are aware, experimental activation energies are not available for the succinimide racemization itself. However, in pH 7 phosphate buffer, the Arrhenius activation energies of Asp racemization were reported to be 21.4, 26.8, and 28.3 kcal mol^−1^ for three synthetic peptides corresponding to parts of human αA-crystallin [21]. On the other hand, the standard enthalpies of the reaction are reported to be about 8 kcal·mol^−1^ for succinimide formation from two Asp-containing peptides (Ace–Asp–Gly–Nme and Ace–Gly–Asp–Gly–Gly–Nme) [15]. Therefore, we can estimate the activation energy of phosphate-catalyzed succinimide racemization to be around 17–18 kcal·mol^−1^, consistent with the calculated activation barrier of 18.8 kcal·mol^−1^ for the model compound. Therefore, the H_2_PO_4_^−^-catalyzed mechanism presently found for succinimide racemization is strongly supported.

## 3. Computational Details

Figure 1 shows the model compound used in the present study (Ace–Asu–Nme), in which an Asu residue is capped with acetyl and NCH_3_ groups on the N- and C-termini, respectively. All calculations were performed by using Spartan ’14 [55]. Geometries were optimized in vacuum by the DFT calculation using the B3LYP functional and the 6-31+G(d,p) basis set. Vibrational frequency calculations were carried out for all the optimized geometries to confirm them either as an energy minimum (with no imaginary frequency) or as a transition state (with a single imaginary frequency) and to correct relative energies for ZPE. The energy minima connected by each transition state were confirmed by IRC calculations, followed by full geometry optimizations. Furthermore, single-point calculations of Gibbs energies of hydration were carried out for the gas-phase optimized geometries by the SM8 continuum model [53,54] at the same level of theory (B3LYP/6-31+G(d,p)).

## 4. Conclusions

A mechanism catalyzed by an H_2_PO_4_^−^ ion has been computationally found for the racemization of the succinimide species formed in proteins and peptides using Ace–Asu–Nme as a model compound. More specifically, the H_2_PO_4_^−^ ion catalyzes enolization of the H_α_–C_α_–C=O portion of the Asu residue by receiving the α proton from C_α_ and donating one of its protons to the carbonyl O atom. The enol, in which the C_α_ atom is sp^2^-hybridized, is the intermediate of succinimide racemization. It has been shown that the initially formed complex between the enol form and H_2_PO_4_^−^ can easily be converted to its mirror-image structure. From there, ketonization of the enol moiety to give the d-form of the Asu residue can proceed catalyzed by the H_2_PO_4_^−^ ion. The calculated activation barrier (18.8 kcal·mol^−1^) was consistent with available experimental energetics, supporting the catalytic mechanism found in the present study. It should also be noted that the barrier is much lower than those calculated for uncatalyzed and water-catalyzed mechanisms [49,56]. Since phosphate buffer is extensively used in in vitro experiments, special care has to be taken when interpreting results of experiments on Asp racemization conducted in phosphate buffer. Moreover, the H_2_PO_4_^−^ ion may also catalyze the in vivo racemization of succinimide intermediates.

## Supplementary Materials

Supplementary materials can be found at www.mdpi.com/1422-0067/17/10/1698/s1.

## References

[1] Geiger T., Clarke S. (1987). Deamidation, isomerization, and racemization at asparaginyl and aspartyl residues in peptides. Succinimide-linked reactions that contribute to protein degradation. J. Biol. Chem..

[2] Stephenson R.C., Clarke S. (1989). Succinimide formation from aspartyl and asparaginyl peptides as a model for the spontaneous degradation of proteins. J. Biol. Chem..

[3] Radkiewicz J.L., Zipse H., Clarke S., Houk K.N. (1996). Accelerated racemization of aspartic acid and asparagine residues via succinimide intermediates: An ab initio theoretical exploration of mechanism. J. Am. Chem. Soc..

[4] Collins M.J., Waite E.R., van Duin A.C.T. (1999). Predicting protein decomposition: The case of aspartic-acid racemization kinetics. Philos. Trans. R. Soc. Lond. B.

[5] Aki K., Fujii N., Fujii N. (2013). Kinetics of isomerization and inversion of aspartate 58 of αA-crystalline peptide mimics under physiological conditions. PLoS ONE.

[6] Takahashi O., Oda A., Jones J.E., Morano D.M. (2012). Amide-iminol tautomerization of the C-terminal peptide groups of aspartic acid residues. Two-water-assisted mechanism, cyclization from the iminol tautomer leading to the tetrahedral intermediate of succinimide formation, and implication to peptide group hydrogen exchange. Tyrosine and Aspartic Acid: Properties, Sources and Health Benefits.

[7] Takahashi O., Kirikoshi R. (2014). Intramolecular cyclization of aspartic acid residues assisted by three water molecules: A density functional theory study. Comput. Sci. Discov..

[8] Takahashi O., Kirikoshi R., Manabe N. (2014). Roles of intramolecular and intermolecular hydrogen bonding in a three-water-assisted mechanism of succinimide formation from aspartic acid residues. Molecules.

[9] Takahashi O., Kirikoshi R., Manabe N. (2015). Acetic acid can catalyze succinimide formation from aspartic acid residues by a concerted bond reorganization mechanism: A computational study. Int. J. Mol. Sci..

[10] Capasso S., Mazzarella L., Sica F., Zagari A. (1989). Deamidation via cyclic imide in asparaginyl peptides. Pept. Res..

[11] Patel K., Borchardt R.T. (1990). Chemical pathways of peptide degradation. II. Kinetics of deamidation of an asparaginyl residue in a model hexapeptide. Pharm. Res..

[12] Patel K., Borchardt R.T. (1990). Chemical pathways of peptide degradation. III. Effect of primary sequence on the pathways of deamidation of asparaginyl residues in hexapeptides. Pharm. Res..

[13] Tyler-Cross R., Schirch V. (1991). Effects of amino acid sequence, buffers, and ionic strength on the rate and mechanism of deamidation of asparagine residues in small peptides. J. Biol. Chem..

[14] Clarke S., Stephenson R.C., Lowenson J.D., Ahern T.J., Manning M.C. (1992). Lability of asparagine and aspartic acid residues in proteins and peptides: Spontaneous deamidation and isomerization reactions. Stability of Protein Pharmaceuticals, Part A: Chemical and Physical Pathways of Protein Degradation.

[15] Capasso S. (1996). Thermodynamic parameters of the reversible isomerization of aspartic residues via a succinimide derivative. Thermochim. Acta.

[16] Shapira R., Austin G.E., Mirra S.S. (1988). Neuritic plaque amyloid in Alzheimer’s disease is highly racemized. J. Neurochem..

[17] Roher A.E., Lowenson J.D., Clarke S., Wolkow C., Wang R., Cotter R.J., Reardon I.M., Zürcher-Neely H.A., Heinrikson R.L., Ball M.J., Greenberg B.D. (1993). Structural alterations in the peptide backbone of β-amyloid core protein may account for its deposition and stability in Alzheimer’s disease. J. Biol. Chem..

[18] Roher A.E., Lowenson J.D., Clarke S., Woods A.S., Cotter R.J., Gowing E., Ball M.J. (1993). β-Amyloid-(1–42) is a major component of cerebrovascular amyloid deposits: Implications for the pathology of Alzheimer disease. Proc. Natl. Acad. Sci. USA.

[19] Fujii N., Ishibashi Y., Satoh K., Fujino M., Harada K. (1994). Simultaneous racemization and isomerization at specific aspartic acid residues in αB-crystallin from the aged human lens. Biochim. Biophys. Acta.

[20] Fujii N., Satoh K., Harada K., Ishibashi Y. (1994). Simultaneous stereoinversion and isomerization at specific aspartic acid residues in αA-crystallin from human lens. J. Biochem..

[21] Fujii N., Momose Y., Harada K. (1996). Kinetic study of racemization of aspartyl residues in model peptides of αA-crystallin. Int. J. Pept. Protein Res..

[22] Iwatsubo T., Saido T.C., Mann D.M.A., Lee V.M.-Y., Trojanowski J.Q. (1996). Full-length amyloid-β(1–42(43)) and amino-terminally modified and truncated amyloid-β42(43) deposit in diffuse plaques. Am. J. Pathol..

[23] Fujii N., Takemoto L.J., Momose Y., Matsumoto S., Hiroki K., Akaboshi M. (1999). Formation of four isomers at the Asp-151 residue of aged human αA-crystallin by natural aging. Biochem. Biophys. Res. Commun..

[24] Lowenson J.D., Clarke S., Roher A.E. (1999). Chemical modifications of deposited amyloid-β peptides. Methods Enzymol..

[25] Orpiszewski J., Schormann N., Kluve-Beckerman B., Liepnieks J.J., Benson M.D. (2000). Protein aging hypothesis of Alzheimer disease. FASEB J..

[26] Kaneko I., Morimoto K., Kubo T. (2001). Drastic neuronal loss in vivo by β-amyloid racemized at Ser^26^ residue: Conversion of non-toxic [d-Ser^26^] β-amyloid 1–40 to toxic and proteinase-resistant fragments. Neurosci..

[27] Fujii N., Matsumoto S., Hiroki K., Takemoto L. (2001). Inversion and isomerization of Asp-58 residue in human αA-crystallin from normal aged lenses and cataractous lenses. Biochim. Biophys. Acta.

[28] Ritz-Timme S., Collins M.J. (2002). Racemization of aspartic acid in human proteins. Aging Res. Rev..

[29] Reissner K.J., Aswad D.W. (2003). Deamidation and isoaspartate formation in proteins: Unwanted alterations or surreptitious signals?. Cell. Mol. Life Sci..

[30] Fujii N. (2005). d-Amino acid in elderly tissues. Biol. Pharm. Bull..

[31] Moro M.L., Collins M.J., Cappellini E. (2010). Alzheimer’s disease and amyloid β-peptide deposition in the brain: A matter of “aging”?. Biochem. Soc. Trans..

[32] Sadakane Y., Konoha K., Kawahara M., Nakagomi K. (2010). Quantification of structural alterations of l-Asp and l-Asn residues in peptides related to neuronal diseases by reversed-phase high-performance liquid chromatography. Chem. Biodivers..

[33] Hooi M.Y.S., Truscott R.J.W. (2011). Racemisation and human cataract. d-Ser, d-Asp/Asn and d-Thr are higher in the lifelong proteins of cataract lenses than in age-matched normal lenses. AGE.

[34] Fujii N., Kawaguchi T., Sasaki H., Fujii N. (2011). Simultaneous stereoinversion and isomerization at the Asp-4 residue in βB2-crystallin from the aged human eye lenses. Biochemistry.

[35] Fujii N., Sakaue H., Sasaki H., Fujii N. (2012). A rapid, comprehensive liquid chromatography-mass spectrometry (LC-MS)-based survey of the Asp isomers in crystallins from human cataract lenses. J. Biol. Chem..

[36] Güttler B.H.-O., Cynis H., Seifert F., Ludwig H.-H., Porzel A., Schilling S. (2013). A quantitative analysis of spontaneous isoaspartate formation from N-terminal asparaginyl and aspartyl residues. Amino Acids.

[37] Tambo K., Yamaguchi T., Kobayashi K., Terauchi E., Ichi I., Kojo S. (2013). Racemization of the aspartic acid residue of amyloid-β peptide by a radical reaction. Biosci. Biotechnol. Biochem..

[38] Inoue K., Hosaka D., Mochizuki N., Akatsu H., Tsutsumiuchi K., Hashizume Y., Matsukawa N., Yamamoto T., Toyo’oka T. (2014). Simultaneous determination of post-translational racemization and isomerization of N-terminal Amyloid-β in Alzheimer’s brain tissues by covalent chiral derivatized ultraperformance liquid chromatography tandem mass spectrometry. Anal. Chem..

[39] Maeda H., Takata T., Fujii N., Sakaue H., Nirasawa S., Takahashi S., Sasaki H., Fujii N. (2015). Rapid survey of four Asp isomers in disease-related proteins by LC-MS combined with commercial enzymes. Anal. Chem..

[40] Fujii N., Takata T., Fujii N. (2015). Quantitative analysis of isomeric (l-α-, l-β-, d-α-, d-β-) aspartyl residues in proteins from elderly donors. J. Pharm. Biomed. Anal..

[41] Fujii N., Takata T., Fujii N., Aki K. (2016). Isomerization of aspartyl residues in crystallins and its influence upon cataract. Biochim. Biophys. Acta.

[42] Takata T., Fujii N. (2016). Isomerization of Asp residues plays an important role in αA-crystallin dissociation. FEBS J..

[43] Konuklar F.A., Aviyente V., Sen T.Z., Bahar I. (2001). Modeling the deamidation of asparagine residues via succinimide intermediates. J. Mol. Model..

[44] Konuklar F.A., Aviyente V., Ruiz-López M.F. (2002). Theoretical study on the alkaline and neutral hydrolysis of succinimide derivatives in deamidation reactions. J. Phys. Chem. A.

[45] Konuklar F.A.S., Aviyente V. (2003). Modelling the hydrolysis of succinimide: Formation of aspartate and reversible isomerization of aspartic acid via succinimide. Org. Biomol. Chem..

[46] Peters B., Trout B.L. (2006). Asparagine deamidation: pH-dependent mechanism from density functional theory. Biochemistry.

[47] Catak S., Monard G., Aviyente V., Ruiz-López M.F. (2006). Reaction mechanism of deamidation of asparaginyl residues in peptides: Effect of solvent molecules. J. Phys. Chem. A.

[48] Catak S., Monard G., Aviyente V., Ruiz-López M.F. (2009). Deamidation of asparagine residues: Direct hydrolysis versus succinimide-mediated deamidation mechanisms. J. Phys. Chem. A.

[49] Takahashi O., Kobayashi K., Oda A. (2010). Modeling the enolization of succinimide derivatives, a key step of racemization of aspartic acid residues: Importance of a two-H_2_O mechanism. Chem. Biodiv..

[50] Tomizawa H., Yamada H., Wada K., Imoto T. (1995). Stabilization of lysozyme against irreversible inactivation by suppression of chemical reactions. J. Biochem..

[51] Bohne C., MacDonald I.D., Dunford H.B. (1986). Measurement of rates and equilibria for keto-enol tautomerism of aldehydes using horseradish peroxidase compound I. J. Am. Chem. Soc..

[52] Bora P.P., Bez G. (2013). Henry reaction in aqueous media at neutral pH. Eur. J. Org. Chem..

[53] Marenich A.V., Olson R.M., Kelly C.P., Cramer C.J., Truhlar D.G. (2007). Self-consistent reaction field model for aqueous and nonaqueous solutions based on accurate polarized partial charges. J. Chem. Theory Comput..

[54] Cramer C.J., Truhlar D.G. (2008). A universal approach to solvation modeling. Acc. Chem. Res..

[55] Wavefunction Inc. (2014). Spartan ’14.

[56] Takahashi O. (2013). Two-water-assisted racemization of the succinimide intermediate formed in proteins. A computational model study. Health.

