# Supplementary material for: Racemization of the Succinimide Intermediate Formed in Proteins and Peptides: A Computational Study of the Mechanism Catalyzed by Dihydrogen Phosphate Ion
Source: Int J Mol Sci. 2016 Oct 10;17(10):1698. doi: 10.3390/ijms17101698 (PMC5085730; doi:10.3390/ijms17101698)
Supplement: Supplementary file 1 [file ijms-17-01698-s001.pdf]

Supplementary Materials: Racemization of the Succinimide Intermediate Formed in Proteins and Peptides: A Computational Study of the Mechanism Catalyzed by Dihydrogen Phosphate Ion

Ohgi Takahashi, Ryota Kirikoshi and Noriyoshi Manabe

Table S1. Total energies (au), zero-point energies (kJ mol^{-1}), and SM8 Gibbs energies of hydration (kJ·mol^{-1}) of the B3LYP/6-31+G(d,p) optimized geometries.

Geometry	Total Energy	Zero-Point Energy	SM8 Gibbs Energy of Hydration
RC	−1251.705747	560.2591	−305.1985290
TS1	−1251.683364	543.8832	−268.8708883
EN1	−1251.698685	555.9825	−254.3189184
TS2	−1251.697719	554.3546	−250.2700462
EN2	−1251.698667	556.2772	−254.2543863
TS3	−1251.698030	555.1928	−257.6665835

Table S2. Cartesian coordinates (Å) of RC (the reactant complex).

Atom	*x*	*y*	*z*
6	1.707089508	−1.335462740	−0.954063235
6	1.384047586	−0.032607614	−0.208850579
6	0.376572094	−0.459014229	0.856323149
8	0.069122745	0.192337609	1.848318390
7	−0.047758843	−1.739227680	0.577082520
6	0.642467602	−2.317991577	−0.486046103
8	0.440092666	−3.442422316	−0.909932812
6	−1.177837998	−2.366235509	1.249740912
1	−1.080539665	−2.213862907	2.327174129
1	−1.158240271	−3.429744002	1.009106018
7	2.492058594	0.636386338	0.464037341
6	3.581363508	1.125543260	−0.189955107
8	3.824121889	0.881211357	−1.371770016
6	4.515762811	1.988778481	0.648585214
1	2.693282776	−1.727713333	−0.685815871
1	5.529935912	1.587613434	0.569767901
1	4.525179638	2.999110088	0.227965343
1	4.231664415	2.045257646	1.703586844
1	1.673399583	−1.232399396	−2.039204752
15	−2.715668901	1.062153280	−0.438865090
8	−3.611761007	−0.262820219	0.091215741
8	−3.636347534	1.943840551	−1.232365751
8	−1.415548164	0.474839941	−0.948948183
1	0.837552215	0.655823151	−0.864046061
8	−2.392113437	1.834234574	0.995272673
1	−1.633076712	1.406398277	1.424801439
1	2.257402587	1.014277959	1.374496572
1	−4.539971528	0.006033933	0.081002627
1	−2.108981608	−1.911037070	0.897577285

Table S3. Cartesian coordinates (Å) of TS1 (the first transition state).

Atom	*x*	*y*	*z*
6	2.191878968	0.223745978	−0.888461560
6	0.974122534	0.457288338	−0.003454161
6	0.917466668	−0.655730396	0.901970119
8	0.224114313	−0.798924681	1.933223555
7	1.752538381	−1.677815794	0.412066717
6	2.522860316	−1.246008379	−0.658470693
8	3.304426492	−1.952086235	−1.284460681
6	1.711282892	−3.043559274	0.898325586
1	1.974089068	−3.079619753	1.959662452
1	2.428949034	−3.621740518	0.313931992
7	0.779071493	1.739074670	0.635294853
6	0.830867718	2.942059806	0.008805421
8	1.238741084	3.096898138	−1.146868427
6	0.368826196	4.132050559	0.842625620
1	3.053824395	0.831052865	−0.578916464
1	1.154371221	4.893597619	0.842710359
1	−0.514877555	4.565420044	0.363190518
1	0.116992514	3.875141192	1.876378622
1	2.035533805	0.413768272	−1.952833681
15	−2.581301371	−0.437991395	−0.380813001
8	−2.419864328	−2.086387897	−0.396454838
8	−3.949571552	−0.033395403	−0.812013726
8	−1.350125963	0.041683445	−1.210649167
1	−0.314367234	0.189223632	−0.720848903
1	0.705061641	−3.455328913	0.773027813
8	−2.337443786	−0.043710124	1.175511480
1	−1.466751832	−0.341003541	1.533896740
1	0.332039200	1.693175347	1.542294175
1	−3.292654646	−2.474325190	−0.246177155

Table S4. Cartesian coordinates (Å) of EN1 (the first enol minimum).

Atom	*x*	*y*	*z*
6	−2.898011954	−0.999585776	0.136925890
6	−1.472002503	−0.511428177	0.057743500
6	−1.490553270	0.839937013	−0.016871269
8	−0.542930444	1.784174240	−0.076274375
7	−2.831174190	1.322658153	−0.012125425
6	−3.724604802	0.283416918	0.076182901
8	−4.951620625	0.392720754	0.093686883
6	−3.198797100	2.719106837	−0.097062639
1	−2.761786694	3.288610889	0.729633099
1	−4.288192113	2.765677588	−0.045613149
7	−0.314462357	−1.306862022	0.135249983
6	−0.249867995	−2.652046349	−0.066646741
8	−1.235500501	−3.357673653	−0.324904489
6	1.140029215	−3.252284087	0.078088984
1	−3.122899437	−1.538258205	1.065515309
1	1.171571907	−3.845965825	0.999368200
1	1.303329312	−3.939619740	−0.756989962
1	1.946967174	−2.514253503	0.103924967
1	−3.181176599	−1.670273270	−0.679447645
15	3.166193170	0.567092638	0.024169731
8	4.005889231	1.597068216	1.012570373
8	3.860065008	−0.757508165	−0.037086037
8	3.301064772	1.298310953	−1.453008209
1	2.665672717	2.023954468	−1.521969503
1	−2.854578337	3.160437963	−1.039000073
8	1.707923651	0.693831963	0.463688773
1	0.376088820	1.402907555	0.117091208
1	0.558726316	−0.796828714	0.298909269
1	4.916556928	1.276555569	1.064613289

Table S5. Cartesian coordinates (Å) of TS2 (the second transition state).

Atom	*x*	*y*	*z*
6	−2.890180749	−0.991417928	0.219316928
6	−1.465419759	−0.516329953	0.073922059
6	−1.477597450	0.832085929	−0.050659476
8	−0.528323513	1.765799946	−0.169372130
7	−2.815830770	1.322343564	−0.027587407
6	−3.711805164	0.292896003	0.125887836
8	−4.937436009	0.409639492	0.170063028
1	0.391121170	1.400884827	0.065378828
6	−3.177278796	2.717478428	−0.155432086
1	−2.730290191	3.311896296	0.648104511
1	−4.265920616	2.772052358	−0.096952915
7	−0.310440824	−1.315952951	0.160956446
6	−0.243552873	−2.650309809	−0.102476699
8	−1.221814160	−3.337405826	−0.429787307
6	1.137464749	−3.265331070	0.067914245
1	−3.088226043	−1.479685365	1.182196671
1	1.145456122	−3.864151240	0.986626253
1	1.310287339	−3.949929655	−0.767358842
1	−3.200837747	−1.699898103	−0.553776244
15	3.142319017	0.585698969	0.046019312
8	1.685749938	0.679676350	0.501876420
8	3.287977684	1.464414581	−1.350620848
1	2.693784781	1.103847901	−2.022556576
1	−2.837566625	3.125163747	−1.113774550
8	3.785848101	−0.766903529	−0.077256514
8	4.039238073	1.553204720	1.025729751
1	0.559119110	−0.811848139	0.362302266
1	4.902808857	1.133096477	1.135626965
1	1.949679085	−2.533483418	0.114414649

Table S6. Cartesian coordinates (Å) of EN2 (the second enol minimum).

Atom	*x*	*y*	*z*
6	−2.892481723	−0.994967343	0.238818868
6	−1.471294354	−0.516856654	0.071935660
6	−1.489176838	0.829431359	−0.070495202
8	−0.545578893	1.764569486	−0.211765020
7	−2.828842106	1.315500769	−0.040156696
6	−3.719532272	0.285260116	0.134988703
8	−4.945347043	0.398134805	0.188621609
1	0.375229980	1.407190319	0.013106938
6	−3.196225226	2.706932811	−0.189140156
1	−2.744873270	3.316782742	0.600293208
1	−4.284581585	2.758942304	−0.123146425
7	−0.311693464	−1.310278119	0.151384621
6	−0.239578631	−2.644867602	−0.107771176
8	−1.218351085	−3.340707449	−0.414685799
6	1.148309591	−3.249263345	0.045324155
1	−3.079746975	−1.468890782	1.211096537
1	1.199700441	−3.782254538	1.002631689
1	1.285375326	−3.988637253	−0.748369453
1	−3.208529114	−1.715709732	−0.520643353
15	3.125974860	0.596742076	0.044104885
8	1.684001062	0.709587379	0.515714930
8	3.323284433	1.648239924	−1.221681127
1	3.716272780	1.147614138	−1.949107007
1	−2.865152498	3.098846605	−1.157041289
8	3.731461423	−0.728573942	−0.338857992
8	4.023225691	1.310692503	1.228790967
1	0.556471029	−0.800194705	0.338403456
1	4.923866334	0.963684896	1.176577638
1	1.958405657	−2.514621397	0.004963646

Table S7. Cartesian coordinates (Å) of TS3 (the third transition state).

Atom	*x*	*y*	*z*
6	−2.928932798	−0.959718310	0.199225703
6	−1.507714881	−0.499847908	−0.017001602
6	−1.513107280	0.848724432	−0.145998346
8	−0.570063922	1.779841446	−0.324938897
7	−2.843022464	1.352405607	−0.049238238
6	−3.739587753	0.333579507	0.156209654
8	−4.959468166	0.463641414	0.273388349
1	0.360882518	1.395660682	−0.267063013
6	−3.196555421	2.751142244	−0.158811820
1	−2.708857520	3.340500536	0.624743100
1	−4.280886948	2.815183281	−0.050606054
7	−0.361184378	−1.314452865	−0.006975853
6	−0.335632380	−2.671437536	−0.085367945
8	−1.348823684	−3.382627015	−0.162082302
6	1.057047475	−3.284516196	−0.062421157
1	−3.084026102	−1.458843280	1.163102369
1	1.108996514	−3.995717199	0.768736567
1	1.198477031	−3.856898608	−0.985616688
1	−3.290688286	−1.655421539	−0.563631929
15	3.210352539	0.566924301	0.070239478
8	1.707411892	0.597411697	−0.143305369
8	3.917952861	1.359353668	−1.203960085
1	4.582698211	0.760655024	−1.569710713
1	−2.895701940	3.156162136	−1.130925079
8	3.946396134	−0.728723110	0.282408762
8	3.495413986	1.609521666	1.316139574
1	0.533028388	−0.812087763	0.000501530
1	4.359632242	1.403055444	1.696649194
1	1.866951105	−2.556697941	0.037520011
